# Nurse-Led Mobile Phone Intervention to Promote Self-Management in Type 2 Diabetes in Ghana: A Randomized Controlled Trial

**DOI:** 10.1177/26350106241293113

**Published:** 2024-11-26

**Authors:** Ernest Asante, Gillian Carter, Helen McAneney, Victoria Bam, Osei Sarfo-Kantanka, Gillian Prue

**Affiliations:** Faculty of Education, Health and Wellbeing, University of Wolverhampton, Wolverhampton, UK; School of Nursing and Midwifery, Queen’s University Belfast, Belfast, UK; School of Nursing and Midwifery, Queen’s University Belfast, Belfast, UK; Northern Ireland Public Health Research Network, School of Medicine, Ulster University, Belfast, UK; School of Nursing and Midwifery, College of Health Sciences, Kwame Nkrumah University of Science and Technology, Kumasi, Ghana; School of Medical Sciences, College of Health Sciences, Kwame Nkrumah University of Science and Technology, Kumasi, Ghana; School of Nursing and Midwifery, Queen’s University Belfast, Belfast, UK

## Abstract

**Purpose:**

The purpose of the study was to test the effectiveness of a nurse-led mobile phone intervention (NMPI) on glycemic variability and self-management among people living with type 2 diabetes (T2DM) in Ghana.

**Methods:**

In this randomized controlled trial, the intervention group received a 3-month NMPI program plus standard care, and the control group received standard care alone in a tertiary health care setting. Ninety-eight participants (baseline A1C > 7%) were randomized 1:1 to either NMPI or standard care group. The primary study outcomes were changes in A1C testing and self-management assessed using the Summary of Diabetes Self-Care Activities tool at baseline and end of the study.

**Results:**

The intervention group had statistically significant improvement in their mean A1C level from baseline to the end of the study. In comparison, the control group also had improvement in their mean A1C level but was not statistically significant. Consistently, the intervention participants had better statistically significant improvements in self-management behaviors than the control group. There was a medium, negative correlation between A1C changes and overall self-care changes for the intervention group, whereas that of the control group was smaller.

**Conclusions:**

Study findings have shown that a tailored NMPI program in addition to standard care could improve glycemic variability and self-management among people living with poorly managed T2DM in Ghana better than standard care alone.

Diabetes mellitus (DM) is a public health problem challenging the Ghanaian health care system, which is already burdened with managing infectious diseases.^1^ The estimated national prevalence is 2% to 9%, most of whom have type 2 DM (T2DM).^2-5^ Diabetes self-management education (DSME) is the cornerstone of effective management and known to increase diabetes-related knowledge and self-care, translating to lower A1C levels and prevention of diabetes complications.^6,7^ Clinical guidelines recommend educating and supporting individuals with T2DM through DSME programs tailored to personal preferences, values, and needs to effectively self-manage, make informed decisions, and increase collaboration with health care professionals.^8-10^ Research has reported the effectiveness of telephone follow-up DSME interventions in promoting self-management and glycemic variability among individuals living with T2DM.^11-13^ However, whereas most of these clinical trials were conducted in developed countries that have better diabetes care expenditures,^2^ little is known about the outcomes of such trials in Sub-Saharan Africa (SSA), which has limited diabetes care resources to compare with.

Ghana’s diabetes care is challenged by limited resources, making it more difficult for nurses to support self-management of patients with T2DM in their homes.^1,14^ In the Ghanaian culture, many people have low health literacy and poor health perceptions; some people with T2DM have misconceptions that diabetes has supernatural causes and is curable through herbal and faith-based treatments.^15-17^ Health care professionals, most of whom are nurses, are perceived as health literacy assets on whom people rely significantly for health education in Ghana.^18^ Thus, nurses providing diabetes care are better placed to deliver diabetes education and self-management follow-up support at patients’ homes, provided alternate avenues, such as via the telephone, could be explored. Fortunately, most Ghanaian adults now have access to mobile phones, compared to the past decade.^19,20^ The present study was based on a successful feasibility trial conducted in 2018,^21^ which demonstrated the feasibility of a mobile phone intervention in following up on T2DM patients in the SSA health care setting. Limitations of this pilot trial included a small sample size, short intervention duration, and some results that were not statistically significant. Therefore, the present study aims to address these limitations and provide further evidence testing the effectiveness of a nurse-led mobile phone intervention on glycemic variability and self-management in Ghana.

## Methods

### Design

This study was a single-blinded parallel-group, two-arm randomized controlled trial (RCT) comparing the effectiveness of a 3-month, nurse-led mobile phone intervention (NMPI) plus usual care with that of only standard care among patients with T2DM. In this type of study design, participants are randomly assigned to 1 of 2 groups (arms), usually an intervention group and a control group. Each group receives its assigned treatment concurrently throughout the study.

### Setting and Recruitment

The trial was conducted at the Diabetes Centre of the Komfo Anokye Teaching Hospital (KATH) in Kumasi, the second-largest urban area in Ghana and a tertiary health care facility, from August 2019 until January 2020. Information on the study setting and eligibility criteria for recruitment have been detailed previously.^21^ Ethical approval was provided by the Research Ethics Committee of the School of Nursing and Midwifery, Queen’s University Belfast and the Committee on Human Research, Publications and Ethics of the School of Medical Sciences, Kwame Nkrumah University of Science and Technology, Ghana, following administrative permit from the Research and Development Unit of KATH. The trial is registered at the Pan African Clinical Trials Registry: PACTR201907488398987 (https://pactr.samrc.ac.za/).

### Sample Size Calculation, Randomization, and Blinding

Sample size calculations were carried out in GPower (version 3.1),^22^ for a 2-tailed *t* test to detect differences in the means of 2 independent groups. With an effect size of 0.63 (based on the pilot study data^21^), α = 5%, 80% power, and assuming 20% attrition (from a similar study^23^), 98 participants are required to be recruited, 49 in each group.

A computerized randomization allocation sequence was generated,^24^ and study participants were assigned at a ratio of 1:1 to either study arm using sealed envelopes after baseline measurements.^25^ Blinding participants or interventionists to the intervention received was not possible due to the nature of the intervention; however, outcome assessors were blinded to the group allocations.

### Nurse-led Mobile Phone Intervention

The NMPI program involved an initial whole-day workshop on diabetes clinical presentation and management education followed by a 3-month follow-up mobile phone calling phase to support practical self-management among the intervention participants only.^21^ The nurse-led calling phase comprised 2 calls per week for the first 4 weeks and then 1 call weekly for the next 9 weeks. Three nurses were involved in the delivery of the calls; therefore, the intervention group was subdivided into 3 cohorts, each rotating every month to a different interventionist to control bias.^26,27^ The nurses received training on the study protocol, intervention delivery processes, and procedures. Over 3 months, the NMPI program was costed by adding the total airtime cost for the 17 calls at 20 minutes per protocol call duration per participant and the total cost of nurses’ time attempting/completing calls per participant.

The transtheoretical model,^28^ which comprises 5 stages of behavior change, theoretically underpins the design of the NMPI components. The initial workshop addressed the precontemplation, contemplation, and preparation stages. The follow-up calls reinforced the preparation stage while focusing more on the practicality of behavior changes at the action and maintenance stages. The NMPI emphasized recognizing change processes at the various stages and responding to them with tailored actions, summarized in Table 1.

**Table 1.** The Transtheoretical Model Applied to the Nurse-Led Mobile Phone Intervention Program

Stage of Change	Process of Change	Assumption and Action (Applying to the Nurse-Led Mobile Phone Intervention Program)
Precontemplation	Consciousness and awareness raising	Assumption: Individuals with suboptimally managed type 2 diabetes may not yet acknowledge the need for behavior change. Health promotion efforts in this stage focus on raising awareness about the risks and consequences of the disease. Action: Discuss diabetes complications and the health problems associated with suboptimal glycemic variability and the feasibility of adhering to diabetes self-management guidelines^9,10^ with individuals. Educate the individual about the importance of healthy eating, physical activity, blood glucose monitoring, foot care, and medication adherence in diabetes care and advantages and disadvantages of being adherent/nonadherent, with diabetes specialists nurses’ practical demonstrations at the initial workshop aided by a locally tailored pictorial booklet^29^ and audiovisual educational resources.
Contemplation	Recognition of the benefits of change	Assumption: Individuals recognize the need for change but may be uncertain or ambivalent about taking action. Health promotion interventions during this stage aim to address any practical patients’ barriers or concerns. Providing educational materials, counseling, and support groups can help patients explore the facilitators and barriers of behavior change, ultimately enhancing their motivation. Action: Discuss with individuals the potential benefits to them, illustrating what diabetes self-management success would mean. Support the individual to identify benefits of adhering to diabetes self-management guidelines^9,10^ and encourage them to develop diabetes self-management plans at the initial workshop.
Preparation	Identification of barriers	Assumption: As individuals progress to this stage, they begin actively planning for behavior change. Health promotion efforts focus on helping patients set realistic goals, develop action plans, and identify potential support systems. Education about healthy lifestyle choices, such as diet modifications and regular exercise, is crucial during this stage. Action: Assist individuals to identify practical barriers they may face and how they might be addressed, including potential support systems. Support any improvements in healthy eating, physical activity, blood glucose monitoring, foot care, and medication adherence in their daily diabetes care. Help identify potential resources to support diabetes self-management at the initial workshop and reinforce during the 13-week nurse mobile phone calls.
Action	Program of change	Assumption: Individuals are actively implementing the behavior change strategies. Health promotion interventions at this stage may include personalized coaching, monitoring progress, and providing positive reinforcement. Action: Develop a plan for adhering to diabetes self-management guidelines with individuals. Support them to identify strategies to address barriers and set and evaluate personalized self-management goals. Encourage self-monitoring of blood glucose, promote adherence to medication regimens, and offer practical exercise and nutrition guidance to facilitate successful behavior change. Support confidence to maintain the change during 13-week nurse mobile phone calls.
Maintenance	Follow-up and continuing support	Assumption: Individuals are continuing their practice of the newly adopted behaviors to last. Health promotion efforts during this stage aim to reinforce positive changes, prevent relapse, and provide ongoing support. Action: Organize regular routine follow-ups and discuss the likelihood of relapse. Encourage individuals to adhere to the diabetes self-management guidelines^9,10,29^ during 13-week nurse mobile phone calls and to maintain the behavior changes during and beyond the intervention delivery period.

To standardize the intervention delivery, a call guide comprising participants’ contact details, call time, and duration logs; self-care activities, including personal goals, action plans, and outcomes for evaluation; and self-care challenges sections was used unchanged as piloted.^21^ Each NMPI participant was allocated 1 call guide, which the interventionists used to record all follow-up calls and weekly self-management progress. All call guide documents were managed and kept securely by the interventionists and were reviewed monthly by 2 investigators (EA and OS) to ensure intervention fidelity and quality control.^30^ The content of the calls included information on diet, exercise, medication taking, self-monitoring of blood glucose, and foot care. The interventionists assisted participants in setting new and evaluating old individualized self-management goals at each call session.

### Current Practice (Usual Care)

All participants report to the Diabetes Centre for their scheduled clinic appointments with their diabetes doctors, nurses, and dietician, which is the current standard care throughout the study period. However, the control group participants were not given any follow-up intervention apart from the usual care.^21^

### Data Collection

Data were collected at baseline and at the end of the intervention by 4 volunteer bachelor-level registered nurses who were blinded to participants’ group allocations. Data collected included blood samples, clinical measurements, and self-reported questionnaires. An independent phlebotomist team blinded to group allocations took all blood samples for A1C (primary outcome) and lipid profile (secondary outcome) testing at the central biochemistry laboratory of KATH (an accredited laboratory adhering to International Organization for Standardization standards [ISO Standard 15,189:2012]).

### Measurements and Instruments

A1C was assessed using the turbidimetric inhibition immunoassay method with a ROCHE COBAS Integra 400 plus analyzer, which is standard practice and has been used in another diabetes trial in Ghana.^31^ Other secondary outcomes measured included systolic and diastolic blood pressure (SBP and DBP), weight, height, body mass index (BMI), and abdominal and hip circumference. The SBP and DBP of participants were measured in a sitting position using an electronic blood pressure monitor. Blood pressure readings were taken 3 times, and the average was recorded. Participants’ BMI was calculated in the standard way using their height and weight. Abdominal and hip circumference were measured with a measuring tape and the participant standing.

Self-reported questionnaires were used to record participants’ demographic characteristics, medical history, and adherence to self-management practices (primary outcome). Self-management was assessed using the revised Summary of Diabetes Self-Care Activities (SDSCA),^32^ which has been used in several diabetes self-management studies.^33-36^ The SDSCA measures the frequency of self-care activities (diet, exercise, blood glucose checking, medication taking, and foot care) over the previous 7-day period, making the assessment of adherence easier by totaling and averaging the various self-care domains. The SDSCA scale has no clinical cutoff for optimal or suboptimal self-care behaviors. However, it provides a range of 0 to 7 days, depicting how often the individual performed those tasks in the previous week. Thus, an overall self-care score was calculated by summing all domains such that higher scores suggest better self-care adherence and vice versa. Studies assessing the psychometric properties of the revised SDSCA scale elsewhere have appraised its content validity and reliability and reported an overall Cronbach’s alpha values of .618 and .735.^35,37^ Similarly, in Ghana, Mogre et al,^38^ reported a Cronbach’s alpha of .68, concluding that the scale is valid and reliable for use in the Ghanaian setting.

### Statistical Analyses

Analysis was conducted using SPSS version 29 for Mac (SPSS Inc, Chicago, IL, USA) software program. Descriptive statistics (mean and standard deviation of interval data; frequency and percentages for categorical data) were used for the demographic and clinical characteristics of the intervention and control groups, including the reporting of missing data. Differences between intervention and control groups at baseline were explored using independent *t* tests and chi-square tests. Data were analyzed on an intention-to-treat (ITT) basis,^39^ using paired *t* tests to detect changes over time (baseline versus end of study) and independent *t* tests to compare the primary and secondary outcomes of the control and intervention groups. ITT is a principle in clinical trials where all participants are analyzed in their initially allocated groups regardless of whether they completed the intervention or adhered to the treatment protocol. This approach preserves the benefits of randomization and provides a more realistic estimate of the treatment’s effectiveness in real-world settings.^39^ Pearson correlation was used to determine the relationship between A1C changes and overall self-care adherence changes. In the ITT analysis, the researchers imputed any missing follow-up measurement data using multiple imputation technique.^40^ Per protocol (as-treated) analysis was performed as a sensitivity analysis to detect any within-group and between-group differences in the groups’ A1C and SDSCA changes. *P* values ≤ .05 were considered statistically significant.

## Results

### NMPI Program Completion Rate, Intervention Fidelity, and Cost

**Figure 1.**
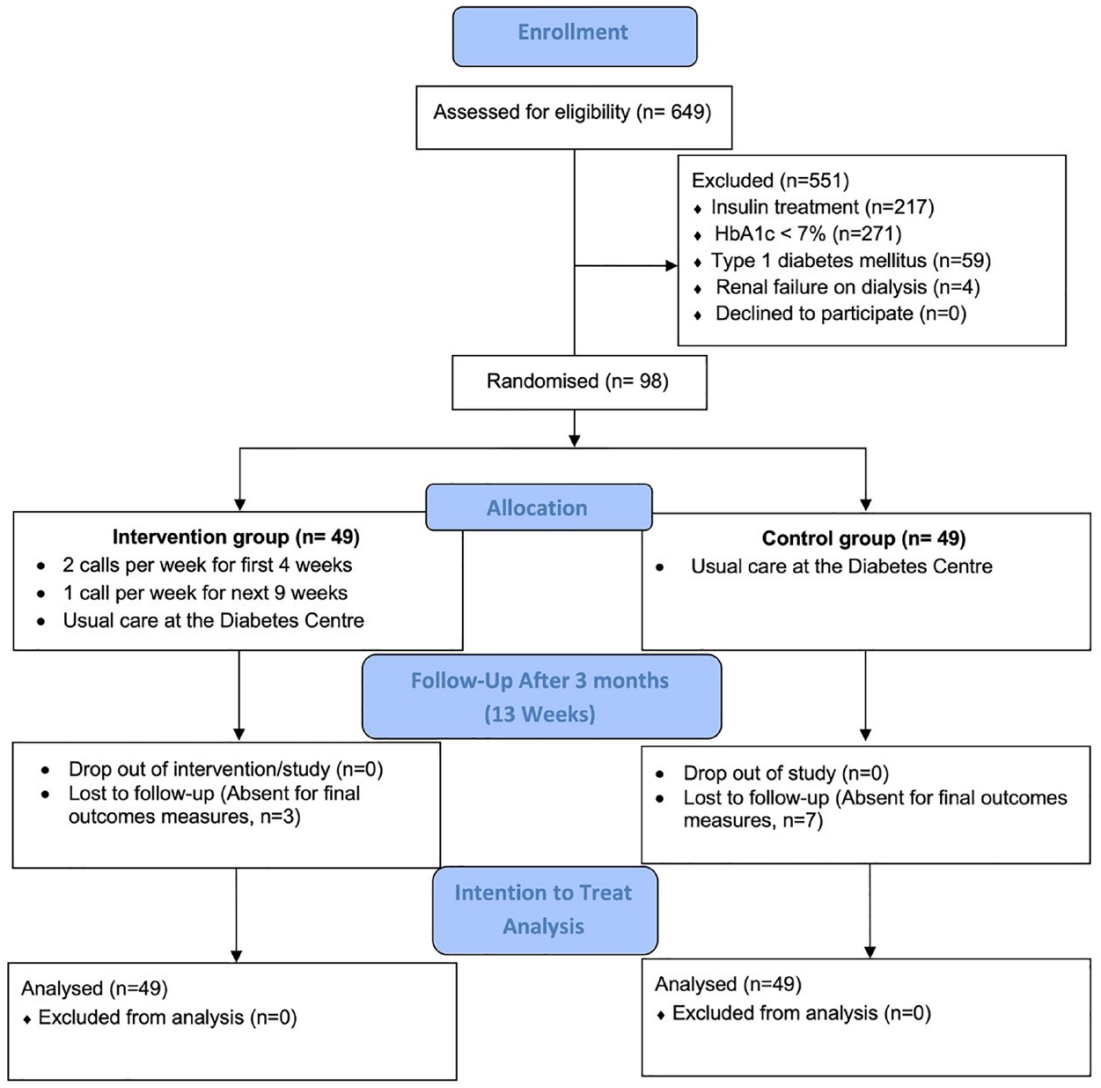
Study flow diagram showing participants’ recruitment, intervention delivery, and analysis numbers.

As shown in Figure 1, the program completion rate was 100%, with no dropouts in the intervention or the control groups. However, 3 intervention and 7 control participants could not attend the end-of-study outcome measurements for personal reasons. Therefore, 88 participants (89.80%) completed end-of-study measurements and self-reported questionnaires (46, 93.88% in the intervention group versus 42, 85.71%, in the control group).

The interventionists attempted/completed all 17 call sessions as scheduled per protocol, each lasting between 1 and 55 minutes. The total weekly call duration across the NMPI participants consistently reduced from week 1 (20 hours: 38 minutes) to week 13 (1 hour: 55 minutes). All NMPI participants completed at least 76.47% of scheduled calls (range: 13 to 17 calls completion per participant), with nearly 60% of participants achieving 100% of scheduled call sessions.

The total airtime cost for 17 calls for 49 participants was GHC 1999.20 (USD 350.74, OANDA Dec 2019), which was GHC 40.8 (USD 7.16) per participant for 3 months. The basic wage for an average nurse in Ghana working 8 hours Monday through Friday during the study period was GHC 12 per hour (USD 2.11, OANDA Dec 2019).^41,42^ Therefore, using the per protocol call duration, each call cost USD 0.70. The 3 nurses’ time for attempting/completing all calls for 49 participants was USD 583.10 for 3 months, which equals USD 11.90 per participant. Therefore, the total cost of nurses’ time and airtime per participant was USD 19.06.

### Baseline Characteristics and Comparison Between the 2 Study Groups

Both intervention and control groups were comparable in baseline characteristics, such as age, diabetes duration, and oral glycemic-lowering medication usage, with no statistically significant differences (Table 2). The mean A1C of the intervention group was 10.14% (SD = 1.88), and that of the control group was 10.28% (SD = 2.24); *P* = .733. Baseline self-management behaviors (SDSCA scoring) regarding diet, exercise, blood glucose monitoring, medication taking, and foot care were similar on average, with no statistically significant differences for both study groups (summarized in Table 3).

**Table 2.** Demographic and Clinical Characteristics of Participants at Baseline

Demographic and Clinical Characteristics	Intervention Group (n = 49)	Control Group (n = 49)	*P* Value
Age (y), mean ± SD (median)	58.71 ± 11.04 (59.0)	57.76 ± 10.96 (59.0)	.667^a^
Gender, n (%)			.443^c^
Female	41 (83.7)	38 (77.6)	
Male	8 (16.3)	11 (22.4)	
Marital status, n (%)			.836^c^
Married	30 (61.2)	29 (59.2)	
Single/divorced/widowed	19 (38.8)	20 (40.8)	
Highest education, n (%)			.459^c^
Basic (primary/middle school/JSS)	37 (75.5)	34 (69.4)	
Secondary	3 (6.1)	6 (12.2)	
Tertiary	8 (16.3)	6 (12.2)	
None	1 (2.0)	3 (6.1)	
Working status, n (%)			.381^c^
Yes	32 (65.3)	36 (73.5)	
No	17 (34.7)	13 (26.5)	
Monthly income (GHC), n (%)			>.999^c^
Below 1000	39 (79.6)	39 (79.6)	
1000-2000	10 (20.4)	10 (20.4)	
Health insurance, n (%)			>.999^c^
Yes	48 (98.0)	48 (98.0)	
No	1 (2.0)	1 (2.0)	
Smoking, n (%)			>.999^d^
Yes	1 (2.0)	0 (0)	
No	48 (98.0)	49 (100.0)	
Alcohol drinking, n (%)			.505^c^
Yes	4 (8.2)	6 (12.2)	
No	45 (91.8)	43 (87.8)	
Diabetes duration (y), mean ± SD (median)	10.65 ± 6.31 (10)	11.24 ± 6.18 (10)	.64^a^
Height (m), mean ± SD (median)	1.61 ± 0.06 (1.61)	1.62 ± 0.08 (1.61)	.508^a^
Weight (kg), mean ± SD (median)	71.60 ± 18.19 (65.0)	72.16 ± 13.37 (74.0)	.862^a^
Body mass index (kg/m^2^), mean ± SD (median)	27.71 ± 7.22 (25.44)	27.45 ± 4.58 (27.25)	.833^a^
Abdominal circumference (cm), mean ± SD (median)	98.59 ± 13.10 (100.0)	97.47 ± 14.49 (98.0)	.688^a^
Hip circumference (cm), mean ± SD (median)	109.02 ± 19.16 (109.0)	105.70 ± 17.04 (106.0)	.367^a^
A1C (%), mean ± SD (median)	10.14 ± 1.88 (10.2)	10.28 ± 2.24 (10.2)	.733^a^
Systolic BP (mmHg), mean ± SD (median)	139 ± 18.75 (139.0)	140.39 ± 17.74 (141.0)	.86^a^
Diastolic BP (mmHg), mean ± SD (median)	83.22 ± 12.63 (83.0)	81.76 ± 12.44 (80.0)	.563^a^
Total cholesterol (mmol/L), mean ± SD (median)	5.29 ± 1.41 (4.98)	5.20 ± 1.21 (5.14)	.912^b^
Triglycerides (mmol/L), mean ± SD (median)	1.59 ± 0.78 (1.50)	1.51 ± 0.80 (1.25)	.308^b^
HDL cholesterol (mmol/L), mean ± SD (median)	1.23 ± 0.46 (1.22)	1.30 ± 0.45 (1.20)	.465^a^
LDL cholesterol (mmol/L), mean ± SD (median)	3.33 ± 1.21 (3.30)	3.22 ± 1.09 (3.15)	.657^b^
Family history of diabetes, n (%)			.113^c^
Yes	32 (65.3)	39 (79.6)	
No	17 (34.7)	10 (20.4)	
Oral hypoglycemic agents, n (%)			
Metformin, n (%)	37 (75.5)	40 (81.6)	0.460^c^
Sulphonylureas, n (%)	26 (53.1)	25 (51.0)	.840^c^
Other oral hypoglycemic agent, n (%)	9 (18.4)	9 (18.4)	>.999^c^
Anti-hypertensive agent users, n (%)	22 (44.9)	27 (55.1)	.312^c^
Lipid-lowering agent users, n (%)	17 (34.7)	22 (44.9)	.302^c^
Diabetes complications, n (%)			
Retinopathy	10 (20.4)	7 (14.3)	.424^c^
Neuropathy	6 (12.2)	17 (34.7)	.009^c^
Foot ulcer	1 (2.0)	1 (2.0)	>.999^d^

Abbreviations: BP = blood pressure; GHC = Ghana cedis (approximately US $0.17, OANDA exchange rate Dec 22, 2019); HDL = high-density lipoprotein; JSS = junior secondary school; LDL = low-density lipoprotein.

a Independent samples *t* test.

b Mann-Whitney U test.

c Chi-square.

d Fisher’s exact test.

**Table 3.** Description of Self-Management Behavior Using Summary of Diabetes Self-Care Activities Questionnaire (Baseline – Follow-Up)

Self-Care Domains (Possible Range: 0-7)	Intervention Group (n = 49)			Control Group (n = 49)			Between-Groups Differences (Mann-Whitney U)	
	Baseline Mean ± SD (Median)	End of Study Mean ± SD (Median)	*P* Value (Wilcoxon Test)	Baseline Mean ± SD (Median)	End of Study Mean ± SD (Median)	*P* Value (Wilcoxon Test)	Baseline *P* Value	End of Study *P* Value
Diet (general and specific)	3.60 ± 1.92 (3.75)	4.99 ± 1.61 (5.0)	<.001	3.37 ± 1.66 (3.25)	4.44 ± 1.49 (4.5)	.003	.474	.448^a^
General diet	3.32 ± 2.81 (3.5)	4.98 ± 2.40 (6.0)	<.001	2.74 ± 2.72 (3.0)	4.47 ± 2.01 (4.4)	.001	.294	.80
Following a healthful eating plan	3.33 ± 2.86 (4)	5.02 ± 2.44 (6)		2.69 ± 2.67 (3)	4.47 ± 2.05 (4.5)			
Following your eating plan over past month	3.31 ± 2.80 (3)	4.93 ± 2.38 (6)		2.71 ± 2.71 (3)	4.47 ± 2.00 (4.4)			
Specific diet	3.87 ± 1.65 (3.5)	5.00 ± 1.51 (5.0)	.001	4.04 ± 1.40 (3.5)	4.40 ± 1.42 (4.4)	.311	.994	.072^a^
Eating 5 or more servings of fruits and vegetable	2.12 ± 2.45 (2)	3.82 ± 2.60 (4)		2.63 ± 2.47 (2)	3.04 ± 2.21 (3)			
Eating high fat foods such as red meat or full-fat dairy products	1.51 ± 2.40 (0)	0.81 ± 1.34 (0)		1.55 ± 1.91 (1)	1.23 ± 1.50 (1)			
Exercise	3.12 ± 2.88 (3.0)	4.75 ± 2.50 (6.0)	.004	3.80 ± 2.93 (4.0)	4.45 ± 2.37 (4.6)	.176	.183	.192
30 mins of physical activity	3.18 ± 2.99 (3)	5.09 ± 2.56 (7)		3.86 ± 2.97 (4)	4.64 ± 2.54 (5)			
Specific physical exercise	3.06 ± 2.90 (3)	4.42 ± 2.72 (5)		3.73 ± 3.01 (3)	4.27 ± 2.65 (4)			
Blood glucose monitoring	0.44 ± 1.11 (0.0)	0.70 ± 1.21 (0.0)	.174	0.42 ± 1.15 (0.0)	0.67 ± 1.41 (0.0)	.243	.826	.905
Testing your blood sugar	0.47 ± 1.17 (0)	0.75 ± 1.26 (0)		0.41 ± 1.15 (0)	0.67 ± 1.41 (0)			
Testing blood sugar the recommended number of times	0.41 ± 1.06 (0)	0.65 ± 1.22 (0)		0.43 ± 1.17 (0)	0.67 ± 1.41 (0)			
Medication	5.67 ± 2.14 (7.0)	6.61 ± 1.17 (7.0)	.004	4.99 ± 2.81 (7.0)	6.29 ± 1.41 (7.0)	.005	.504	.814
Taking your recommended diabetes medication type	5.69 ± 2.15 (7)	6.70 ± 1.06 (7)		5.27 ± 2.81 (7)	6.38 ± 1.40 (7)			
Taking your recommended number of diabetes pills	5.65 ± 2.15 (7)	6.53 ± 1.46 (7)		4.71 ± 3.05 (7)	6.20 ± 1.50 (7)			
Foot care	4.61 ± 1.75 (5.4)	5.66 ± 1.63 (5.6)	.009	4.68 ± 1.79 (5.2)	5.34 ± 1.75 (5.6)	.045	.917	.564
Feet examination	4.22 ± 2.90 (5)	5.88 ± 2.35 (7)		3.69 ± 3.20 (4)	4.82 ± 2.71 (7)			
Inspecting the inside of your shoes	3.80 ± 2.99 (5)	5.90 ± 2.30 (7)		3.65 ± 3.22 (4)	5.03 ± 2.55 (7)			
Feet washing	5.33 ± 2.55 (7)	6.28 ± 1.90 (7)		5.53 ± 2.76 (7)	5.69 ± 2.28 (7)			
Soaking your feet	3.18 ± 3.06 (3)	3.00 ± 3.38 (0)		2.20 ± 3.06 (0)	1.68 ± 2.63 (0)			
Drying between toes after washing	5.06 ± 2.73 (7)	6.25 ± 1.94 (7)		5.22 ± 2.91 (7)	5.84 ± 2.17 (7)			
Overall self-care (all domains combined: 0-119)	55.92 ± 17.20 (59.0)	72.40 ± 12.81 (76.0)	<.001	55.29 ± 16.77 (55.0)	67.26 ± 14.53 (67.0)	<.001	.854^a^	.268^a^

a Independent samples *t* test.

### End of Study Measurements Compared (ITT Analysis)

#### Within-group differences: glycemic variability (A1C) and self-management behavior (SDSCA)

The NMPI group had statistically significant improvement in their mean A1C level from 10.14% (SD = 1.88) to 9.15% (SD = 2.09; *P* = .005) at the end of the study (−0.98%; 95% CI, −1.64 to −0.31). Most of these participants (69.4%, n = 34) had improvements, whereas 30.6% (n = 15) had worsening A1C levels at the end of the study compared to baseline. Among the intervention participants with A1C improvements, only 16.3% (n = 8) were optimal (<7%),^9^ whereas that of the majority (83.7%, n = 41) remained suboptimal (>7%). In comparison, the control group also had improvement in their mean A1C level (10.28%, SD = 2.24 to 9.80%, SD = 2.19; *P* = .113; −0.48%; 95% CI, −1.09 to 0.12) but did not reach statistical significance. Many of the control participants either had their A1C levels worsen (46.9%, n = 23) or did not have any changes (4.1%, n = 2), with 49.0% (n = 24) having some improvements. The majority (91.8%, n = 45) continued to have suboptimal A1C; only 8.2% (n = 4) achieved optimal level.

Consistently, as detailed in Table 3, the intervention group reported statistically significant improvements in their self-management behaviors in days/week for overall diet (*P* < .001), general diet (*P* < .001), specific diet (*P* = .001), exercise (*P* = .004), medication taking (*P* = .004), and foot care (*P* = .006) but not blood glucose monitoring (*P* = .174) at the end of the study. Their overall self-care rose from 55.92 (SD = 17.20) at baseline to 72.40 (SD = 12.81; *P* < .001) at the end of the study. Pearson correlation found a medium, negative correlation between A1C changes and overall self-care changes, which was statistically significant (*r* = −.459, N = 49, *P* = .001) for the intervention group.

Although lesser in comparison to the intervention group, the control group also reported statistically significant improvements in their self-management behaviors regarding overall diet (*P* = .003), general diet (*P* = .001), medication taking (*P* = .005), and foot care (*P* = .045) but not adherence to specific diet (*P* = .311), exercise (*P* = .176), and blood glucose monitoring (*P* = .243) guidelines at the end of the study. The overall self-care average for this group rose from 55.29 (SD = 16.77) at baseline to 67.26 (SD = 14.53; *P* < .001) at the end of the study. There was, however, a small, negative correlation between A1C changes and overall self-care changes, which was not statistically significant (*r* = −0.164, n = 49, *P* = .260) for this study group.

#### Between-group differences: glycemic variability (A1C), self-management behavior (SDSCA) and other clinical characteristics

The intervention participants achieved a greater reduction in mean A1C values than the control participants (−0.98% vs −0.48%; *P* = .272) at the end of the study. Similarly, the intervention group had higher self-management scores in all areas (diet: *P* = .80; exercise: *P* = .192; blood glucose monitoring: *P* = .905; medication: *P* = .814; foot care: *P* = .564; overall self-care: *P* = .268) compared to that of the control group, but none of these were statistically significant. Presented in Table 4, the between-group differences for changes in the secondary outcomes were marginal and did not reach statistical significance.

**Table 4:** Changes in Clinical Characteristics Within and Between Groups^a^

	Intervention Group (n = 49)				Control Group (n = 49)				Between-Groups Mean Difference
	Baseline Mean ± SD (Median)	End of Study Mean ± SD (Median)	Mean Change (95% CI)	*P* Value	Baseline Mean ± SD (Median)	End of Study Mean ± SD (Median)	Mean Change	*P* Value	*P* Value
A1C (%)	10.14 ± 1.88 (10.2)	9.16 ± 2.09 (9.1)	−0.98 (−1.64 to −0.31)	.005^b^	10.28 ± 2.24 (10.2)	9.80 ± 2.19 (9.5)	−0.48 (−1.09 to 0.12)	.116^b^	.272^d^
Systolic BP	139.73 ± 18.75 (139)	139.47 ± 20.17 (140)	−0.27 (−6.46 to 5.93)	.799^c^	140.39 ± 17.74 (141)	137.84 ± 17.81 (136)	−2.55 (−7.66 to 2.56)	.294^c^	.568^d^
Diastolic BP	83.22 ± 12.63 (83)	86.31 ± 12.82 (84)	3.08 (−0.48 to 6.64)	.133^c^	81.76 ± 12.44 (80)	86.20 ± 11.37 (86)	4.45 (0.05 to 8.85	.072^c^	.629^d^
Total cholesterol (mmol/L)	5.29 ± 1.41 (4.98)	4.64 ± 1.27 (4.48)	−0.65 (−1.00 to −0.30)	<.001^b^	5.20 ± 1.21 (5.14)	4.46 ± 0.94 (4.5)	−0.74 (−0.99 to −0.49)	<.001^b^	.672^e^
Triglycerides (mmol/L)	1.59 ± 0.78 (1.50)	1.27 ± 0.58 (1.16)	−0.32 (−0.54 to −0.10)	<.001^c^	1.51 ± 0.80 (1.25)	1.28 ± 0.66 (1.24)	−0.23 (−0.40 to −0.05)	.013^c^	.210^e^
HDL cholesterol (mmol/L)	1.2292 ± 0.461 (1.22)	1.2335 ± 0.348 (1.20)	0.004 (−0.11 to 0.12)	.941^b^	1.30 ± 0.44 (1.20)	1.22 ± 0.32 (1.26)	−0.076 (−0.19 to 0.04)	.181^b^	.073^e^
LDL cholesterol (mmol/L)	3.33 ± 1.21 (3.30)	2.83 ± 1.06 (2.94)	−0.50 (−0.83 to −0.17)	.004^b^	3.22 ± 1.09 (3.15)	2.66 ± 0.78 (2.67)	−0.56 (−0.79 to −0.33	<.001^b^	.768^e^
Body mass index (kg/m^2^)	27.70 ± 7.22 (25.44)	28.31 ± 7.32 (26.40)	0.77 (−0.20 to 1.40)	.002^c^	27.45 ± 4.58 (27.25)	29.38 ± 4.74 (28.76)	0.96 (0.81 to 3.05)	<.001^c^	.498^d^
Abdominal circumference (cm)	98.59 ± 13.10 (100.0)	95.78 ± 12.43 (95.0)	−2.82 (−4.99 to −0.64)	.012^b^	97.47 ± 14.49 (98.0)	98.52 ± 10.68 (99.0)	1.05 (−2.62 to 4.71)	.568^b^	.275^e^
Hip circumference (cm)	109.02 ± 19.16 (109.0)	103.12 ± 17.91 (103.0)	−5.90 (−9.51 to −2.29)	.001^c^	103.12 ± 17.04 (106.0)	107.78 ± 11.85 (110.0)	2.07 (−2.49 to 6.64)	.689^c^	.049^e^

BP = blood pressure; HDL = high-density lipoprotein; LDL = low-density lipoprotein

a Mean change from baseline to end of study (95% Confidence Interval [CI]) [E.g., −0.1 (−2.7 to −13.6)].

b Paired samples *t* test.

c Wilcoxon signed ranks test.

d Independent samples *t* test.

e Mann-Whitney U test.

### Sensitivity Analysis: Per Protocol (as Treated) Analysis

The per protocol analysis involved only participants for both study groups who attended end-of-study measurements (intervention: n = 46 vs control: n = 42) and consistently showed greater A1C and overall self-care improvements in the intervention group than control group. The NMPI group had statistically significant improvement in their mean A1C level from 10.22% (SD = 1.89) to 9.16% (SD = 2.12; *P* = .004) at the end of the study, representing −1.05% (95% CI, −1.74 to −0.36). Most of these participants (69.6%, n = 32) had improvements, whereas 30.4% (n = 14) had worsening A1C levels at the end of the study compared to baseline. In comparison, the control group also had improvement in their mean A1C level (10.24%, SD = 2.31 to 9.71%, SD = 2.31; *P* = .113), representing −0.54% (95% CI, −1.20 to 0.13), but did not reach statistical significance. Many of the control participants either saw their A1C worsen (47.6%, n = 20) or did not see any changes (4.8%, n = 2), with 47.6% (n = 20) having some improvement in their A1C levels. There were marginal differences in overall self-care for both groups (intervention: 55.92, SD = 17.20 to 72.40, SD = 12.81, *P* < .001 vs control: 55.29, SD = 16.77 to 67.26, SD = 14.53, *P* < .001). However, between-group A1C and overall self-care differences were not statistically significant at *P* = .281 and *P* = .193 respectively.

## Discussion

The intervention reported an A1C improvement of approximately 1%, which was twice that seen in usual care. This is clinically significant if maintained given that according to research, a 0.8%-point reduction in A1C could reduce the chances of patients with T2DM developing microvascular complications by 30% and a 17% reduction in diabetes-related mortalities.^43,44^ The intervention improved adherence to diet, physical activity, blood glucose monitoring, medication, and foot care guidelines more than usual care alone. Similar trials delivering intensive telephone coaching self-management support programs for 3 to 4 months have reported similar trends, in which A1C level and adherence to diet, physical activity, and medication improved in the intervention group compared to their usual care counterparts.^13,45,46^

There were no between-group differences in glycemic-lowering medication usage over the study period to account for improvements in glycemic management. Therefore, it is likely that the intervention effect was attributed to self-management behavior changes made by the participants, which is known to lead to significant A1C reductions.^47,48^ The statistically significant negative correlation found between A1C and overall self-care among the NMPI participant supports this inference that as patients improved their adherence to self-management guidelines, they experienced subsequent improvements in their glycemic variability.

Comparing the NMPI program with a similar intervention^49^ shows some common features, such as interventionists being trained diabetes educators under the supervision of senior clinicians; tailored session content focusing on patient empowerment through goal setting and evaluation; negotiation of an action plan that includes appropriate diet, exercise, lifestyle adjustments, diabetes clinic or medical visits, and medication adherence; and the provision of an educational booklet adapted to the local culture. These follow-up support features are additional roles that are absent or limited in current standard diabetes care but nonetheless relevant in engaging and supporting diabetes patients with poor glycemic management in the Ghanaian setting.

The overall cost of the calling phase of the NMPI program is USD 934 and may need central government funding to be delivered nationally. However, spending USD 19.06 to cover nurses’ time and airtime for 3 months to promote glycemic variability and self-management and prevent diabetes complications such as hypoglycemia or hyperglycemia in a patient with poorly managed diabetes is more cost-effective than the USD 22.61 daily hospitalization cost if this patient is admitted and treated for these complications in Ghana.^50^

The intervention appeared to be welcomed and accepted by the participants due to their high engagement levels, completing over 76% of scheduled call sessions with no dropouts. The interventionists collectively achieving 100% call initiation rate demonstrates intervention fidelity that is consistent with that of the pilot.^21^ This affirms the feasibility and acceptability of the novel nurse-led mobile phone calls as a follow-up intervention to support practical T2DM self-management in Ghana. However, there were slight but not significant changes in the lipid profile across the study participants. This could be due to the trial not being sufficiently long to produce a significant effect on blood lipids, as has been reported in previous studies in Africa.^51,52^ Thus, future research could consider a lengthy study period to effectively assess this impact.

The sustainability of the self-management improvements gained through the NMPI program posttrial has yet to be assessed. The benefits of a lifestyle intervention may be short-lived among individuals with T2DM^53^ due to the chronicity of the disease requiring lifelong treatments. Thus, patients may need sustained support and encouragement from clinicians to maintain lifestyle changes, particularly among those who already have been struggling with poor glycemic variability and self-management. In behavioral research, individuals’ motivation to persist in behavior needed to sustain intervention benefits could be reduced after intervention withdrawal and may need ongoing follow-up to facilitate the longer-term maintenance of intervention gains.^54^

Strengths of our study include a randomized design and having a usual care group as control, which limited confounding factors by balancing between-group baseline characteristics, ensuring that the endpoint difference could be attributed to the study treatment received. The study’s internal validity is another strength; all outcome measurements were performed by a single laboratory blinded to the study groups. The acceptance of the NMPI program by the participants and their clinicians and the high intervention fidelity resulting from the quality monitoring ensured that the intervention could be delivered as originally planned. Furthermore, the monthly rotation of nurses across the intervention cohorts increased the robustness of the trial by limiting interventionist attachment and biases.^26^

This trial has some limitations. It provided patients with healthy eating, exercise, medication adherence, and behavioral support but did not collect day-to-day records of these parameters. Therefore, the study did not determine the physiological mechanisms mediating the effects of the intervention. Additionally, patients’ adherence to self-management guidelines was assessed from self-reported data, which is prone to inaccuracies due to recall bias.^55^ The interventionists and participants could not be blinded to study groups. Thus, it is possible that knowledge of group allocation influenced outcomes through favorable expectations associated with randomization to the experimental group.^56^ The trial recruited participants from 1 site, excluding all patients with T2DM who had optimal glycemic variability (A1C < 7). Therefore, to ensure the generalizability of the trial findings, a multicenter trial including all individuals with diabetes is required in future studies.

## Conclusion

To conclude, this RCT has further extended the evidence on the effectiveness of a nurse-led mobile phone intervention in an SSA health care setting. The trial has shown that a tailored NMPI program in addition to standard care could improve glycemic variability and self-management among people living with poorly managed T2DM in Ghana, which is better than standard care alone. Future research could explore the sustainability of these improvements among diabetes populations adhering to treatment guidelines in other developing countries to inform diabetes health care professionals, researchers, policymakers, and stakeholders.
